# High levels of pathological jaundice in the first 24 hours and neonatal hyperbilirubinaemia in an epidemiological cohort study on the Thailand-Myanmar border

**DOI:** 10.1371/journal.pone.0258127

**Published:** 2021-10-07

**Authors:** Laurence Thielemans, Pimnara Peerawaranun, Mavuto Mukaka, Moo Kho Paw, Jacher Wiladphaingern, Jordi Landier, Germana Bancone, Stephane Proux, Henrike Elsinga, Margreet Trip-Hoving, Borimas Hanboonkunupakarn, Tha Ler Htoo, Thaw Shee Wah, Candy Beau, Francois Nosten, Rose McGready, Verena I. Carrara

**Affiliations:** 1 Shoklo Malaria Research Unit, Mahidol-Oxford Tropical Medicine Research Unit, Faculty of Tropical Medicine, Mahidol University, Mae Sot, Thailand; 2 Neonatology-Pediatrics, Université Libre de Bruxelles, Brussels, Belgium; 3 Mahidol-Oxford Tropical Medicine Research Unit (MORU), Faculty of Tropical Medicine, Mahidol University, Bangkok, Thailand; 4 Centre for Tropical Medicine and Global Health, Nuffield Department of Medicine, University of Oxford, Oxford, United Kingdom; 5 IRD, INSERM, SESSTIM, Aix Marseille University, Marseille, France; 6 Department of Medicine, Swiss Tropical and Public Health Institute, Basel, Switzerland; Menzies School of Health Research: Charles Darwin University, AUSTRALIA

## Abstract

Population risks for neonatal hyperbilirubinaemia (NH) vary. Knowledge of local risks permits interventions that may reduce the proportion becoming severe. Between January 2015 and May 2016, in a resource-limited setting on the Thailand-Myanmar border, neonates from 28 weeks’ gestation were enrolled into a prospective birth cohort. Each neonate had total serum bilirubin measurements: scheduled (24, 48, 72 and 144 hours of life) and clinically indicated; and weekly follow up until 1 month of age. Risk factors for developing NH were evaluated using Cox proportional hazard mixed model. Of 1710 neonates, 22% (376) developed NH (83% preterm, 19% term). All neonates born <35 weeks, four in five born 35–37 weeks, and three in twenty born ≥38 weeks had NH, giving an overall incidence of 249 per 1000 livebirths [95%CI 225, 403]. Mortality from acute bilirubin encephalopathy was 10% (2/20) amongst the 5.3% (20/376) who reached the severe NH threshold. One-quarter (26.3%) of NH occurred within 24 hours. NH onset varied with gestational age: at a median [IQR] 24 hours [24, 30] for neonates born 37 weeks or prematurely *vs* 59 hours [48, 84] for neonates born ≥38 weeks. Risk factors for NH in the first week of life independent of gestational age were: neonatal G6PD deficiency, birth bruising, Sgaw Karen ethnicity, primigravidae, pre-eclampsia, and prolonged rupture of membranes. The genetic impact of G6PD deficiency on NH was partially interpreted by using the florescent spot test and further genotyping work is in progress. The risk of NH in Sgaw Karen refugees may be overlooked internationally as they are most likely regarded as Burmese in countries of resettlement. Given high levels of pathological jaundice in the first 24 hours and overall high NH burden, guidelines changes were implemented including preventive PT for all neonates <35 weeks and for those 35–37 weeks with risk factors.

## Introduction

Neonatal hyperbilirubinemia (NH), characterized by elevated levels of total serum bilirubin (TSB) in the blood, is a common condition in neonates. An incorrect or delayed diagnosis may put neonates at risk of developing neurologic dysfunction such as acute bilirubin encephalopathy or a range of subtle to severe kernicterus spectrum disorders [1, 2]. Resource-limited settings endure most of the burden worldwide as public awareness, access to care, and outpatient follow up are often limited: at least 75% of the affected infants reside in sub-Saharan Africa and South Asia [3].

The risk of developing NH depends not only on the physiologic status of the neonate at birth and maternal and environmental factors but also parents’ care-seeking behaviour, health care service monitoring tools, and practice management [4]. Timely recognition and treatment maximize the likelihood of NH remaining uncomplicated. The challenge is to identify neonates at risk of NH as early as possible to plan optimal monitoring.

The National Institute for Health and Care Excellence (NICE) NH guideline [5] has been effective in decreasing the number of neonates with severe NH (13% in 2009–2011) and NH mortality on the Thailand-Myanmar border [6]. This is a resource-limited setting with a high prevalence of glucose-6-phosphate dehydrogenase (G6PD) deficiency: 13.7% in adult males and 2–4% in adult females [7, 8].

This study aims to describe the epidemiology of NH in the aforementioned setting in an actively screened birth cohort from 28 weeks gestation, including: incidence, mortality, time of NH onset by gestational age, and factors associated with NH in the first week of life. This information will identify if any adaptation of the current international guidelines is needed.

## Methods

### Study area and population

This prospective observational cohort study included live births between January 2015 and May 2016 in facilities run by the Shoklo Malaria Research Unit (SMRU). These facilities serve marginalized Karen and Burmese populations, comprising migrants and refugees. Free maternal and child healthcare is offered and includes pre- and postnatal care, and a birthing unit staffed by skilled birth attendants [9]. Low income and education, and poor health conditions prevail [10–12]. GA is estimated either by ultrasound (US) at first antenatal consultation (ANC) or by Dubowitz gestational assessment for late attenders [13, 14]. Women needing caesarean section (no elective procedures) are referred to a local hospital. Following the WHO recommendations, delayed cord clamping is routine unless the neonate requires urgent resuscitation [15]. Most neonates are nursed on the post-natal ward but if an elevated level of neonatal care is required e.g. phototherapy, intravenous antibiotics, nasogastric feeding or oxygen, they are transferred to the Special Care Baby Unit (SCBU), another department within the SMRU facilities [16].

### Inclusion criteria

Any liveborn neonate ≥28 weeks’ gestation was included if seen within 48 hours (h) of birth, or if presenting with jaundice in the first week of life, as homebirth still occurs in this setting.

### Follow up

Follow up in the first week of life included TSB, haematocrit (HCT), and weight; and it was deemed ‘complete’ if a minimum of 3 TSB measurements were available: I) one TSB before or at 30 h of life, II) one TSB measured at a maximum of 36 h after the first one, and III) one TSB between 120 and 168 h (5–7 day of life). An additional TSB was done if the second TSB was performed between 31 to 60 h to ensure that no more than 60 h spanned between measurements taken before 120 h of life. The neonate was considered NH-free between 120 h and 168 h if the TSB measured at 120 h of life was at least 50 μmol/l below the treatment threshold.

Neonates were then followed up (clinical exam, weight, visual assessment of jaundice, and TSB if clinically indicated) every week until one month of age [17]. Mothers were encouraged to bring unwell children to the clinics any time in-between scheduled appointments for examination and treatment.

### Definitions

#### Neonatal hyperbilirubinemia

The NICE hour-specific treatment thresholds based on GA at birth were used to determine NH requiring phototherapy (PT) [5]. NH was defined as moderate when at least one TSB was on or exceeding the PT threshold and severe if at least one TSB was on or exceeding the exchange transfusion threshold. NH diagnosed within the first 48 h of life was categorised as ‘early NH’, while NH occurring between 48–168 h of life was defined as ‘late NH’. The 48 hours cut-off was based on the median duration stay in the postnatal ward for a mother-child dyad with uncomplicated delivery in this setting.

Type, timing, and duration of PT were recorded. Light intensity was measured with a digital light meter (Lightmeter by Medical Technology Transfer and Services Ltd; measuring range 0 to 150 μW/cm^2^/nm; accuracy ± 2%) prior to starting phototherapy conditions were optimized to have the best light intensity possible depending on the severity of the NH [18]. TSB measurement frequency while receiving PT and treatment discontinuation followed the recommendations of the NICE guidelines [5]. The site medical doctor and the head of the department discussed care of each neonate requiring exchange transfusion prior to transfer to the local Thai Hospital in Mae Sot (60 km away) as the procedure was not available in the clinics. A diagnosis of acute bilirubin encephalopathy was considered in the presence of acute onset of neurological signs such as seizure or opisthotonos.

#### Cohort variables

Cohort characteristics were grouped into four categories: maternal, obstetric, neonatal characteristics and clinical events.

Available known risk factors associated with an increased risk of developing NH, as well as characteristics specific to the study site: migrant or refugee status; use of naphthalene balls; neonate G6PD status, and ethnicity; were included. The latter was based on both parents and grandparent’s ethnicity and reported as “mixed” when parents’ ethnicity differed. Of note, people of Islamic faith self-identified as “Burmese Muslim”.

Maternal body mass index (BMI) before delivery was calculated using the last weight within 2 weeks of delivery [19]; a BMI ≥27.5 mg/kg^2^ was categorized as overweight according to the WHO expert consultation on Asian populations [20]. Neonatal resuscitation was defined as the provision, at minimum, of positive pressure ventilation via bag and mask [21]; birthweight was valid if measured within the first 72 hours of life, and typically in the first 30 minutes [22]. Weight-for-gestational age adjusted for sex was calculated according to Interbio-21 international standards and categorized as small for GA (SGA) if lower than the 10^th^ percentile [23].

Clinical events other than neonatal hyperbilirubinemia were SCBU admission and three events occurring within the first week of life but prior to NH: excessive weight loss defined as ≥7% difference between birth weight and subsequent weights [24, 25]; polycythaemia was defined by proxy as capillary HCT ≥70% as the only onsite measure available; and clinically diagnosed severe infections such as sepsis, meningitis, necrotizing enterocolitis or pneumonia, treated with a minimum of five days of intravenous antibiotics.

### Blood sampling

G6PD fluorescent spot test, ABO & rhesus blood group determination, and direct Coombs test were measured on cord blood or venous blood at time of first visit for neonates born outside SMRU birthing unit. The ABO incompatibility definition was limited to neonates with A or B blood type born to type O mothers [26]. TSB and HCT measurements were done by capillary heel prick. This blood sample was centrifuged three minutes at 10 000 rotations per minute to separate red blood cells from plasma; HCT was first estimated using a Hawksley micro-haematocrit reader, then the sample was used to assess TSB using a micro-bilirubinometer (Pfaff Medical Bilimeter 2 and 3). G6PD fluorescent spot test was by double blind read at birth, repeated at one month, and the neonate final G6PD status amended according to the test result obtained at one month [27].

### Statistical analysis

Statistical analyses were performed using SPSS 23.0 (IBM SPSS Statistics, IBM Corporation) and STATA 14 (Stata Corp 2015, Version 14.1. College Station, Texas, StataCorp LP).

Moderate and severe NH incidence was calculated by survival analysis using data from neonates who had a complete regular follow up in the first week of life as defined in the Methods. One TSB per neonate with complete regular follow up measured at, or the nearest to 24 h (12–30 h), 48 h (31–60 h), 72 h (61–80 h) and 144 h (81–168 h) was used to describe TSB levels trend. Only TSB measurements prior to the initiation of PT were included. Time-point TSB median values were compared using Kruskal-Wallis Test.

Potential risk factors for developing NH were evaluated using a Cox proportional hazard mixed model, clustered by site. Variables with p-value <0.2 in univariable analysis, as well as risk-factors for NH described in the literature, were included in a multivariable model, and adjusted for confounding factors. Variables presenting obvious correlation (e.g., maternal age and primigravida, primigravida and history of jaundice in sibling, potential ABO incompatibility and Coombs test, absence of delayed cord clamping and the need of resuscitation or the place of delivery, gender and G6PD deficiency in the neonate, or refugee or migrant status and ethnicity) were not included simultaneously in the models. Analysis of potential risk factors for developing early and late NH excluded neonates who developed late and early NH, respectively.

### Ethics approval and consent to participate

The study was approved by Oxford Tropical Research Ethics Committee, UK (OxTREC 41–144), the Mahidol University Faculty of Tropical Medicine Ethical Committee, Thailand (TMEC 14–012) and the Tak Province Border Community Ethics Advisory Board (TCAB-08-13). Informed consent was obtained from the parent or guardian of the neonate: written for those who were literate or by thumbprint in the presence of a literate witness for illiterate.

## Results

**Fig 1:**
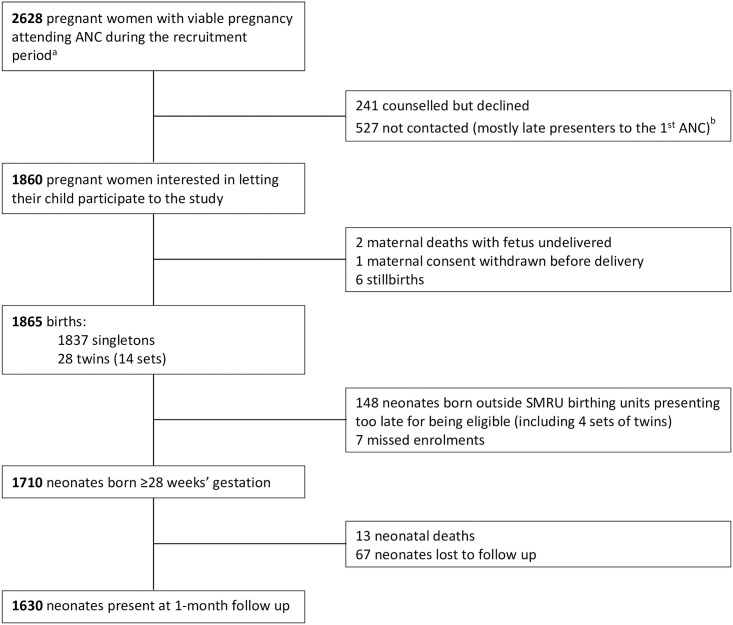
Enrolment flowchart procedure. ^a^Pregnancy outcome before 28 weeks of gestation was considered as a miscarriage [28]. ^b^Demographic characteristics of pregnant women not contacted or who declined were similar to that of pregnant women counselled and interested except for a higher proportion of smokers (13.1% (100/768) *vs* 9.9% (184/1860), p = 0.019).

There were 1710 neonates included of 2628 women attending the facility during the study period, and 95.3% (1630/1710) completed the one-month follow up (Fig 1).

Relevant cohort characteristics are presented in Table 1. Nearly half the population were migrants 53.1% (908/1710) not refugees, with 27.1% (464/1709) of mothers of young maternal age (<20 years), one in three were primigravid and 35.3% (604/1710) were illiterate. Birth included 92.9% (n = 1589) spontaneous vaginal delivery, 5.0% (86/1710) caesarean section and 2.0% (35/1710) instrumental delivery. Delayed cord clamping, 84.5% (1298/1536) was routine for births in the SMRU facilities where most births occurred 89.9% (n = 1536) but not at home 3.6% (n = 61) or in other hospitals/clinics 6.6% (n = 113). The mean birthweight ± standard deviation was 3001 ± 420 grams (n = 1699). Nearly all neonates, 96.6% (1651/1710), were breastfed from birth, 20.4% (346/1699) were SGA, and 4.9% (84/1710) were premature (minimum GA in this cohort was 29^+3^ weeks^+days^). One in three neonates were transferred from the post-natal ward to the SCBU during their first week of life for a median [IQR] 5 days [2, 7]: 8.3% (142/1710) had severe infections, 26.0% (441/1699) weight loss ≥7%, and 14.9% (253/1702) polycythaemia (Table 1).

**Table 1:** Maternal, obstetrical, neonatal characteristics and clinical events.

Characteristics		Neonates born ≥28 weeks’ gestation n = 1710
**Maternal characteristics**		
Literacy		1106 (64.7)
Primigravida		590 (34.5)
Pre-eclampsia or eclampsia		43 (2.5)
Rhesus negative		3/1702 (0.2)
**Obstetric characteristics**		
Rupture of membranes ≥18 h		110/1566 (7.0)
Delayed cord clamping		1354 (79.2)
**Neonatal characteristics**		
Birth bruising or haematoma		60/1689 (3.6)
Ethnicity	Burman	337/1660 (20.3)
	Sgaw Karen	644/1660 (38.8)
	Poe Karen	128/1660 (7.7)
	Burmese Muslim	87/1660 (5.2)
	Others and mixed ethnicity	464/1660 (28.0)
Gestational age	<35 weeks	22 (1.3)
	35–36^+6^ weeks^+days^	62 (3.6)
	37–37^+6^ weeks^+days^	124 (7.3)
	≥38 weeks	1502 (87.8)
Gender (male)		890 (52.1)
Birthweight (g), mean ± SD^a^		3001 ± 420
Breastfeeding		1651 (96.6)
G6PD deficiency (by FST)		120/1708 (7.0)
ABO incompatibility		264/1708 (15.5)
Rhesus incompatibility		3/1700 (0.2)
Positive Coombs test		49/1505 (3.3)
Hypoalbuminemia (<3 g/dl)		5/1512 (0.3)
**Clinical events in first week of life**		
Severe infection		142 (8.3)
Polycythaemia (HCT ≥70%)		253/1702 (14.9)
Weight loss ≥7%		441/1699 (26.0)

Data are presented as n (%).

FST, Fluorescent spot test; G6PD deficiency, glucose-6-phosphate dehydrogenase deficiency; HCT, Haematocrit.

^a^ Birthweight n = 1699.

While a range of other variables were examined: HIV 0.1% (2/1710), syphilis 0.6% (10/1710), smoking 9.3% (158/1709), overweight before delivery 23.9% (399/1671), twins 1.2% (n = 20), use of oxytocin 11.8% (198/1677), being born small for gestational age 20.4% (346/1699), use of naphthalene for storing the clothes 5.4% (92/1710), resuscitation at birth 3.5% (57/1710), sibling with a history of jaundice 16.7% (187/1120), admission to SCBU in the first week of life 31.2% (533/1710); they are not further presented as they did not feature significantly in later analysis, or as per methods were collinear with another variable.

**Fig 2:**
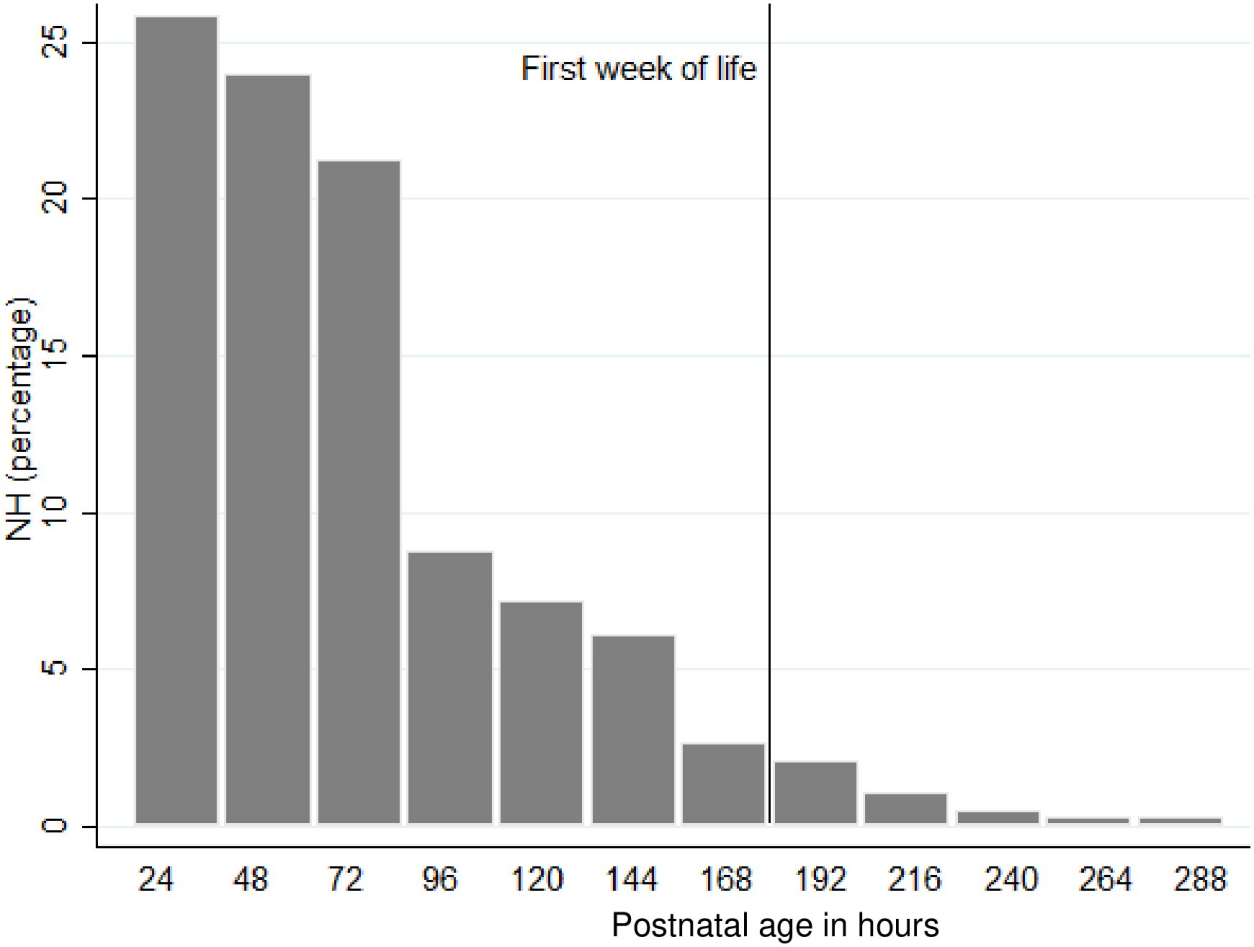
Age of onset at first Neonatal hyperbilirubinemia (NH) episode among 376 neonates born ≥28 weeks of gestation.

NH was diagnosed in 22.0% (376/1710) of neonates at a median [IQR] postnatal age of 50 hours [24, 83]: 95.7% (360/376) in the first week of life and 4.3% (16/376), all moderate, in the second week of life (>168 h postnatal age) (Fig 2). NH occurred in 83.3% (70/84) of preterm and 18.8% (306/1626) of term neonates.

The severe NH threshold was reached by twenty neonates (5.3%, 20/376) and three received exchange transfusion at the referral hospital. Seven cases were severe at first diagnosis (one preterm and 6 term neonates), while 13 developed severe NH after a first diagnosis of moderate NH (3 preterm and 10 term neonates). A third (7/20) of neonates with severe NH were from Sgaw Karen ethnicity. Severe NH mortality was 10% (2/20) and due to acute bilirubin encephalopathy in term neonates: one, born at home arrived in a critical condition and died before referral, the other died despite having received three exchange transfusions.

### NH incidence in the first week of life and by gestational age

**Fig 3:**
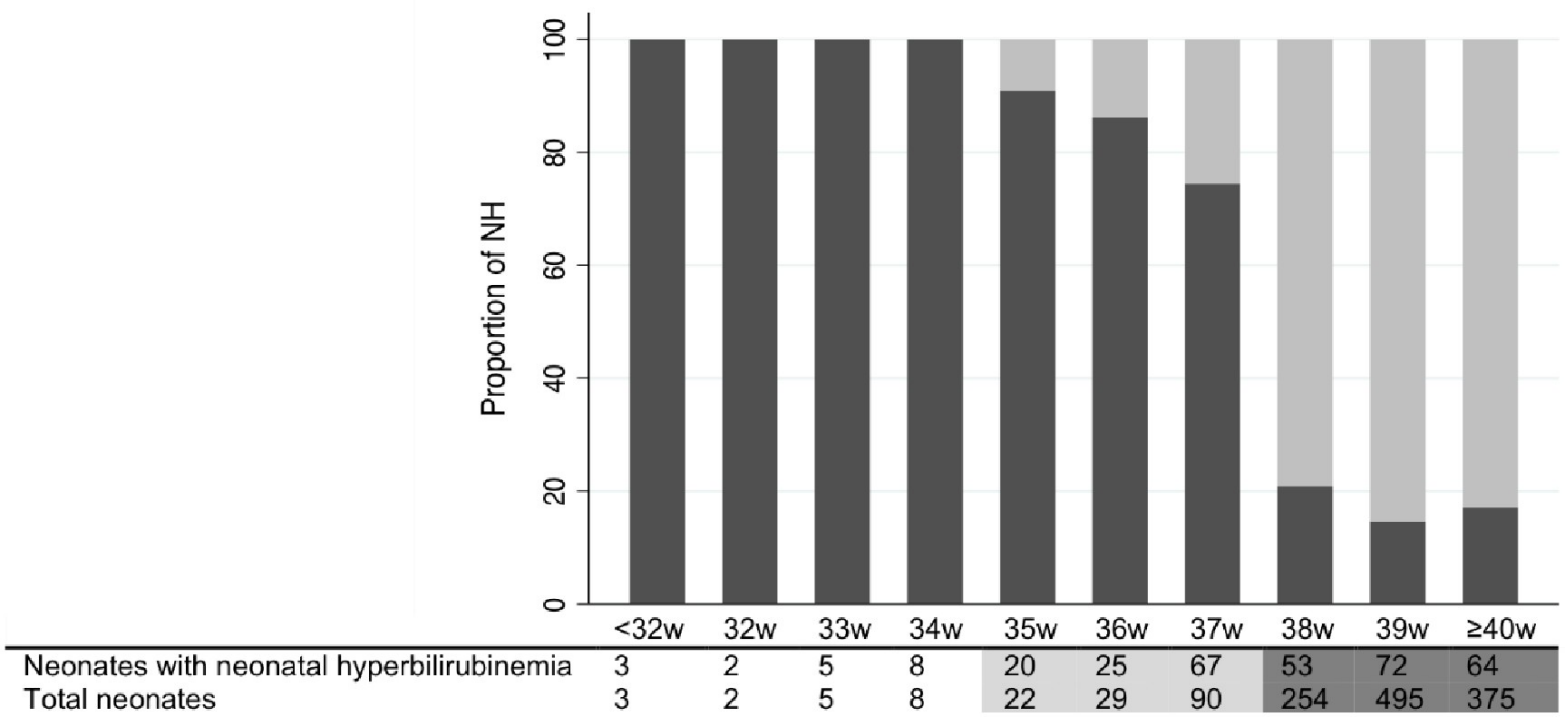
Proportion of neonatal hyperbilirubinemia per gestational age in weeks among 1283 neonates with a complete and regular TSB follow up in the first week of life.

**Fig 4:**
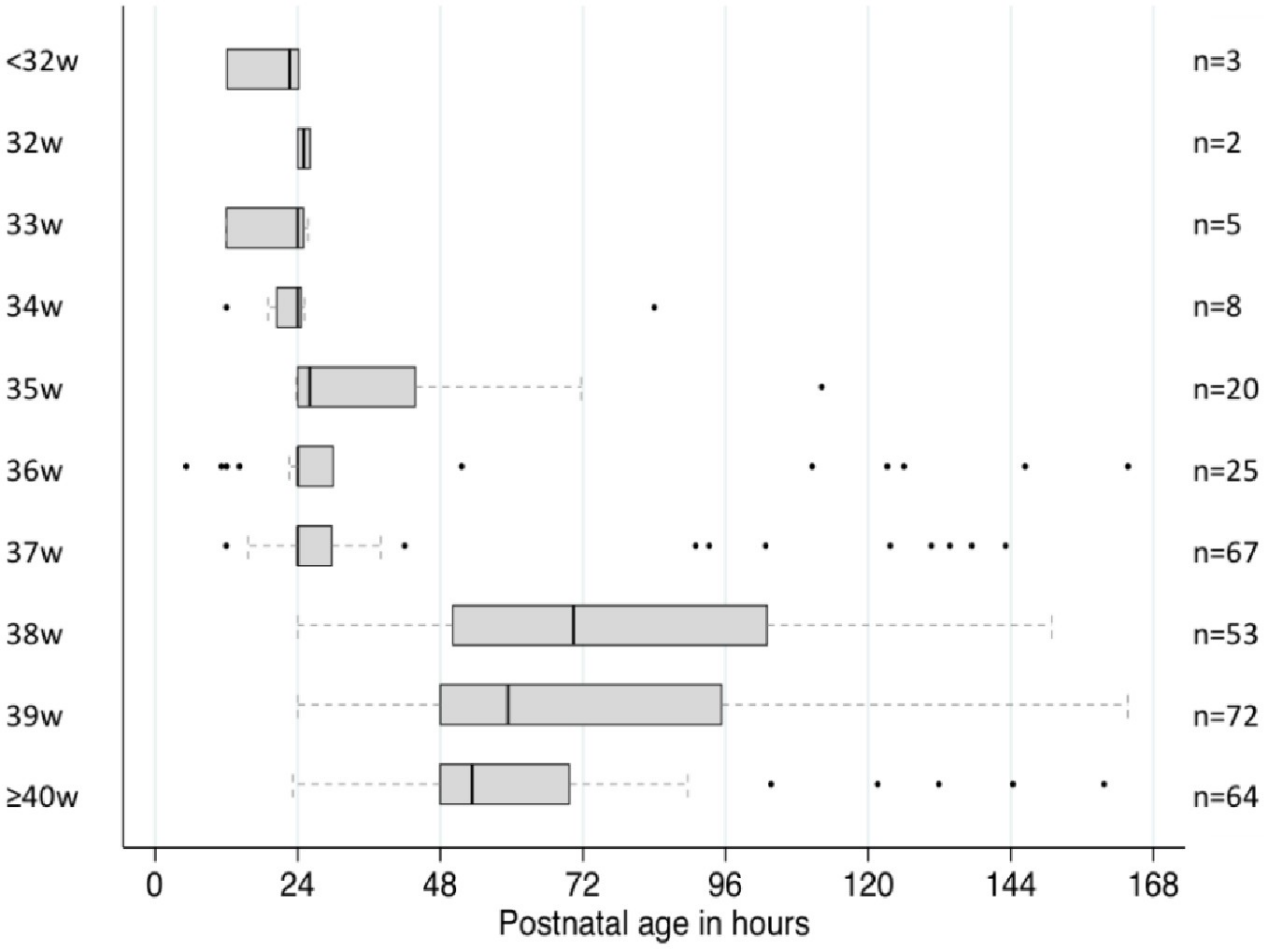
Median postnatal age at onset of neonatal hyperbilirubinemia per gestational age in weeks among 1283 neonates with a complete and regular TSB follow up in the first week of life. Each boxplot delineates the 25^th^ and 75^th^ quartiles with the median represented by the straight bold line. The dotted line defines the upper and lower range and outliers are represented by the dots.

Neonates born by caesarean section in referral hospitals and late presenters did not meet the definition for the sub-cohort of regular complete follow up in the first week of life as they missed the required windows of TSB measurements. Three-quarters (1283/1710) of the cohort including 84.8% of the neonates with NH (319/376) with regular, complete follow up were available, giving a NH incidence in the first week of life of 249 [95%CI 225, 273] per 1000 livebirths (319/1283) and a severe NH incidence of 12 [95%CI 7, 19] per 1000 livebirths (15/1283). Gestational age was important to the prevalence of NH: 91.3% (63/69) born <37 weeks of gestation *vs* 16.8% (189/1124) born ≥38 weeks, p <0.001; with the proportion at 37 weeks of gestation, 74.4% (67/90), more consistent with NH proportions in preterm neonates (Fig 3). Gestational age was also important to the timing of NH with onset at a median [IQR] 24 h [24, 30] for both neonates born 37 weeks (n = 67) or prematurely (n = 63) *vs* 59 h [48, 84] for neonates born ≥38 weeks (Fig 4). Overall, 26.3% (84/319) of NH occurred within 24 h; 58.5% (76/130) neonates with NH and born <38 weeks developed NH within 24 h; and 90.5% (76/84) neonates who developed NH within 24 h of life were those born <38 weeks of gestation (including 84% (n = 64) neonates born 35–37 weeks).

### TSB levels trend

Neonates born at a gestational age ≤34 weeks were excluded from this part of the analysis as all of them developed NH prior to or at the 24 h time-point measurement. Based on the prevalence and time of onset of NH, neonates were categorized as: born at a gestational age 35–37 weeks or ≥38 weeks.

Table 2 presents a comparison of TSB median [IQR] values at 24 h, 48 h and 72 h of life between neonates ≥38 weeks who later develop NH or not, and between those G6PD deficient or not. Both neonates who would later develop NH and those G6PD deficient had significantly higher median TSB values (μmol/L) at each time-point (S1 Fig). At 144 h [85, 168] of life, TSB values of G6PD deficient neonates were still significantly higher (250 [173, 283], (n = 37) *vs* 201 [154, 248], (n = 821), p = 0.011).

**Table 2:** TSB median [IQR] levels at 24 h, 48 h, and 72 h of life in neonates G6PD deficient or not and in neonates who would later develop NH or not among a cohort of neonates born at a gestational age ≥38 weeks and with a complete regular follow up in the first week of life.

Time points in hours (min-max)	TSB^a^, μmol/L			TSB^a^, μmol/L		
	G6PD normal	G6PD deficient	p-value	Did not develop NH	Developed NH	p-value
24 h (15–30) (n = 1111)	113 [99–129] (n = 1038)	131 [116–149] (n = 73)	<0.001	110 [96–124] (n = 935)	140 [126–154] (n = 176)	<0.001
48 h (>30-<60) (n = 1026)	170 [145–193] (n = 973)	186 [164–219] (n = 53)	0.002	166 [143–189] (n = 935)	212 [186–231] (n = 91)	<0.001
72 h (≥60–84) (n = 808)	205 [171–238] (n = 770)	229 [173–259] (n = 38)	0.033	203 [169–235] (n = 774)	273 [251–293] (n = 34)	<0.001

Data are presented as median and [IQR].

^a^ TSB values in absence of NH prior to or at the time of measurement.

Among neonates born 35–37 weeks of gestation, only a minimal number of TSB measurements prior to developing NH were available for each time-point and all were from G6PD normal neonates. At 24 h the median [IQR] TSB was similar for neonates with or without subsequent NH (99 [92, 105] μmol/L, (n = 29) *vs* 98 [88, 108] μmol/L, (n = 29), p = 0.793). On the other hand, 48 h and 72 h median [IQR] TSB values were significantly higher among neonates who would later develop NH: 162 [150, 168] μmol/L, (n = 16) *vs* 138 [126, 146] μmol/L, (n = 29) at 48 h, p = 0.009 and 212 [198, 230], (n = 13) *vs* 169 [153, 190], (n = 28) at 72 h, p <0.001 respectively; the same trend as observed for neonates ≥38 weeks.

### Factors associated with an increased risk to develop NH

The general characteristics of neonates NH-free and those of neonates with early NH (within 48 h of life) or with late NH (48–168 h of life) are presented in S1 Table. Factors associated with an increased risk to develop NH in the first week (168 h) of life other than gestational age were G6PD deficiency, HR 3.89, [95%CI 2.81, 5.38]; presence of birth bruising or haematoma, HR 2.10, [95% CI 1.31, 3.37]; Sgaw Karen ethnicity, HR 1.45, [95%CI 1.11, 1.89]; primigravid mothers, HR 1.74, [95%CI 1.37, 2.2]; presence of pre-eclampsia or eclampsia, HR 1.41, [95%CI 1.09, 1.82]; and prolonged rupture of membranes, HR 1.93, [95%CI 1.32, 2.83], S2 Table. Utilizing the same model structure, polycythaemia at 24 h of life was an additional significant risk factor for early NH (48 h) (HR 1.75, [95%CI 1.05, 2.91]), Table 3. Maternal pre-eclampsia or eclampsia, prolonged rupture of membranes and birth bruising or haematoma were not associated with an increased risk of late NH (Table 4).

**Table 3:** Uni- and multivariable analysis of potential risk factors for developing early NH (in 48h of life) using Cox proportional hazard mixed model clustering by site, n = 1136.

Characteristics	Neonates without NH in the first week of life (n = 964)^a^	Neonates with NH in the first 48 hours (n = 172)	Univariable analysis		Multivariable analysis^b^	
			HR [95% CI]	p-value	HR [95% CI]	p-value
**Maternal Characteristics**						
Literacy (cannot read)	346 (36)	59 (34)	0.92 [0.67, 1.27]	0.618		
Primigravida	296 (31)	80 (47)	1.97 [1.46, 2.67]	<0.001	1.70 [1.22, 2.38]	0.002
Pre-eclampsia or eclampsia	15 (2)	14 (8)	4.78 [2.76, 8.28]	<0.001	1.58 [1.19, 2.10]	0.002
**Obstetric characteristics**						
Rupture of membranes ≥18 h^c^	52/951 (5)	18/165 (11)	2.07 [1.27, 3.39]	0.004	2.03 [1.22, 3.40]	0.006
Delayed cord clamping	854 (89)	136 (79)	0.51 [0.35, 0.73]	<0.001	0.88 [0.56, 1.37]	0.572
**Neonatal Characteristics**						
Gestational age (<38 weeks)	29 (3)	111 (65)	38.20 [27.90, 52.20]	<0.001	38.90 [28.00, 54.00]	<0.001
Birth bruising or haematoma^c^	29/ 963 (3)	11 (6)	2.13 [1.15, 3.92]	0.016	2.53 [1.30, 4.92]	0.006
Ethnicity (Sgaw Karen)^c^	351/939 (37)	80/169 (47)	1.49 [1.10, 2.01]	0.010	1.53 [1.10, 2.12]	0.011
Gender (male)	482 (50)	102 (59)	1.44 [1.06, 1.95]	0.018		
G6PD deficiency (by FST)	41 (4)	30 (17)	4.24 [2.86, 6.28]	<0.001	5.03 [3.29, 7.70]	<0.001
ABO incompatibility	138 (14)	37 (22)	1.63 [1.14, 2.35]	0.008	1.30 [0.89, 1.90]	0.174
Positive Coombs test^c^	31/937 (3)	10/157 (6)	1.90 [1.01, 3.62]	0.050		
**Clinical events in first 24 h of life**						
Severe infection at 0–24 h of life	39 (4)	14 (8)	2.09 [1.21, 3.62]	0.008	1.14 [0.62, 2.11]	0.674
Weight loss ≥7% at 24 h [12–30 h] of life	23 (2)	7 (4)	1.72 [0.81, 3.68]	0.158		
Polycythaemia (HCT ≥70%) at 24 h [12–30 h] of life	81 (8)	20 (12)	1.46 [0.91, 2.33]	0.116	1.75 [1.05, 2.91]	0.030

Data are presented as n (%).

^a^ Neonates who develop late NH (n = 147) were excluded from the analysis.

^b^ Adjusted for Primigravida, Pre-eclampsia or eclampsia, Rupture of membrane ≥18 h, Delayed cord clamping, Gestational age <38 weeks, Birth bruising or haematoma, Sgaw Karen ethnicity, G6PD deficiency (by FST), ABO incompatibility, Severe infection 0-24h, and Polycythaemia. Gender and Positive Coombs test were not included because they were highly correlated with G6PD deficiency (by FST), and ABO incompatibility, respectively.

^**c**^ Rupture of membranes ≥18 h n = 1116, Birth bruising or haematoma n = 1135, Ethnicity (Sgaw Karen) n = 1180, Positive coombs test n = 1094.

**Table 4:** Uni- and multivariable analysis of potential risk factors for developing late NH (48–168 h of life) using Cox proportional hazard mixed model clustering by site, n = 1111^a^.

Characteristics	Univariable analysis		Multivariable analysis^b^	
	HR [95% CI]	p-value	HR [95% CI]	p-value
**Maternal Characteristics**				
Literacy (cannot read)	0.79 [0.56, 1.12]	0.187	0.80 [0.55, 1.16]	0.237
Primigravida	1.66 [1.19, 2.30]	0.003	1.68 [1.18, 2.39]	0.004
Pre-eclampsia or eclampsia	0.84 [0.21, 3.41]	0.812		
**Obstetric characteristics**				
Rupture of membranes ≥18 h^c^	1.47 [0.81, 2.66]	0.201		
Delayed cord clamping	0.69 [0.44, 1.06]	0.093	0.96 [0.59, 1.55]	0.869
**Neonatal Characteristics**				
Gestational age (<38 weeks)	3.66 [2.26, 5.92]	<0.001	4.60 [2.76, 7.67]	<0.001
Birth bruising or haematoma^c^	2.52 [1.36, 4.66]	0.003	1.82 [0.92, 3.61]	0.088
Ethnicity (Sgaw Karen)^c^	1.66 [1.12, 2.46]	0.012	1.65 [1.11, 2.44]	0.013
Gender (male)	1.32 [0.95, 1.83]	0.097	NA	
G6PD deficiency (by FST)	2.85 [1.76, 4.61]	<0.001	3.20 [1.93, 5.31]	<0.001
ABO incompatibility	1.09 [0.69, 1.72]	0.704		
Positive Coombs test^c^	0.61 [0.19, 1.92]	0.398		
**Clinical events in first 24 h of life**				
Severe infection at 0–24 h of life	1.75 [0.92, 3.32]	0.089		
Weight loss ≥7% at 24 h [12–30 h] of life	1.38 [0.57, 3.37]	0.479		
Polycythaemia (HCT ≥70%) at 24 h [12–30 h] of life	1.15 [0.65, 2.04]	0.631		

^a^ Neonate who developed early NH (n = 172) were excluded from this analysis.

^b^ Adjusted for Literacy, Primigravida, Delayed cord clamping, Gestational age <38 weeks, Presence of birth bruising or haematoma, Sgaw Karen ethnicity, G6PD deficiency (by FST). Gender was not included as it was highly correlated with G6PD deficiency (by FST).

^**c**^ Rupture of membranes ≥18 h n = 1258, Birth bruising or haematoma n = 1282, Ethnicity (Sgaw Karen) n = 1250, Positive coombs test n = 1234.

## Discussion

This study conducted on the Thailand-Myanmar border, in a setting where neonatal care but not intensive neonatal care is available, reports a high NH incidence during the first week of life (249 per 1000 livebirths) in neonates born from 28 weeks’ gestation. One in five neonates in this setting required phototherapy for NH at median [IQR] of 50 h [24–83]. The increase in NH from 18.5% previously reported from this setting (2009 to 2014) [6] to 22% in this study, most likely results from the systematic TSB screening from 24 hours of life onwards including follow up to the end of the first month of life. Severe NH is 25 times higher than that observed in high-income countries (11 *vs* 0.4 per 1000 livebirths [29]), although systematic TSB screening is the most likely reason for its decline from 14.9% in 2011 [6] to 5.3% during this study.

Systematic TSB screening and access to PT using the high-income country NICE international guidelines has been a positive effort towards reducing neonatal mortality as recommended under the global strategy of the Every Newborn Action Plan (ENAP) [30] but the study findings also indicate the guidelines are not optimal for this setting. UK, as well as the American guidelines propose that TSB should not be done routinely unless the neonate is visibly jaundiced in first 24 h of life or jaundice appears excessive for the age [5, 31]. Jaundice can be visualized at TSB values between 85–100 μmol/L [32] but it may be more difficult in darker skin neonates, particularly preterms, and those <35 weeks would likely reach the PT threshold prior to having a TSB measurement done. The UK guidelines recommend NH cases within the first 24 h of life be referred to tertiary hospital for medical review, while neonates <38 weeks of gestation, those exclusively breastfed or with a previous sibling who had NH should remain in close monitoring for 48 hours—an impractical recommendation in a setting where nearly all neonates are exclusively breastfed from birth and access to referral care is extremely limited. Care for preterm neonates born <35 weeks of gestation, representing 5% of livebirths in this cohort, is not included in the American guidelines and although the PT thresholds are detailed for each gestational week from 23 until 37 in the NICE guidelines, there is no specific guidance for monitoring NH in those <35 weeks.

Several of the study findings are challenging to solve for a resource-limited setting and could be relevant to other health centres caring for born-too-soon and born-too-small neonates. As far as we are aware this study represents the first report of a high proportion (26%) of pathological NH in the first 24 hours of life in a resource-limited setting: all preterm neonates <35 weeks of gestation developed NH, and all within the first 24 h. The high rate of NH in four of five neonates born at 35 to 37 weeks of gestation, half of whom developed this within 24 h, is a more unusual pattern but is partially recognized by both American Academy of Pediatrics and the NICE guidelines. Premature neonates are at higher risk of developing severe NH and developing acute neurological sequelae [33]. Early screening is therefore paramount to avoid delaying the start of PT and thus reducing the risk of bilirubin-induced neurological dysfunction; as in this study, the value of the first TSB screening done at 24 h of life or earlier was already above the gestational-age adjusted PT threshold in nearly all preterm neonates. PT within the first 24 h has shown to be effective in reducing NH among preterms [34] and those born 28 to 34 weeks of gestation could probably benefit from low irradiance prophylactic PT [35]. Concerns of increased mortality in extremely low birth weight infants under PT [36] are not as relevant in this setting where mechanical ventilation is not available and survival below <28 weeks of gestation is unlikely [28]. However, it is impractical to offer prophylactic PT to all neonates born at 35 to 37 weeks’ and tools to assist in identification of those with high risk to develop NH, such as the use of a non-invasive cord blood TSB measurement [37], are being tested at the Shoklo Malaria Research Unit clinics, as a possible method to detect rising TSB levels at the earliest stage.

The 18.8% of NH among term neonates observed in this study represents a heavy burden: twice the estimates of 1 in 10 neonates requiring phototherapy annually worldwide [32]. This cohort was closely monitored, and this high prevalence is not explained because families presented late for TSB check as may be expected in a LMIC setting. This places a significant workload and intensity of TSB monitoring that is uncomfortable (multiple heel pricks because transcutaneous devices are beyond LMIC resources) for neonates and their families. Furthermore, NH occurs at a median [IQR] time of 59h [48-84h] in neonates born 38 weeks or more, and this has clinical repercussions. Otherwise healthy term neonates are usually discharged within 24–48 hours in a setting like the one on the Thailand-Myanmar border and due to transportation and access difficulties parents are not always able to return for timely follow-up.

Severe NH was observed in this setting after a first diagnosis of moderate NH. This highlights the need for constant vigilance once PT has started and for tools that improve detection of risk including among term infants.

Apart from prematurity and G6PD deficiency there were four independent risk factors specific to an increased risk of developing early NH, within the first 48 hours of life: maternal pre-eclampsia or eclampsia, prolonged rupture of membranes, both recognized in a systematic literature review on maternal risk factors for NH [38], a high neonatal haematocrit and the presence of birth bruising or haematoma. Of these factors, only birth bruising or haematoma is listed as major risk factor by the American Academy of Pediatrics guidelines for closer monitoring [18]. One additional context-specific factor was recognized: neonates of Sgaw Karen ethnicity showed an increased risk to develop NH in the first week of life compared to the other ethnic groups. A study in 105 neonates of different Asian ethnicities proposed differences in genes involved in bilirubin metabolism or environmental factors as a possibility for NH readmission [39]. In this setting, Sgaw Karen have a higher frequency of G6PD deficiency [7] and analysis of genetic factors involved in bilirubin metabolism is ongoing. Knowledge of an increased NH risk in Sgaw Karen ethnicity is valuable locally in Thailand and Myanmar, and internationally given that more than 100 000 Sgaw Karen refugees from the Thailand-Myanmar border, most likely regarded as Burmese, now reside in at least 14 countries, following the largest UNHCR relocation programme globally [40].

Based on the results from the current study, a decision to modify the current guidelines using a combination of TSB levels and risk factors was implemented, aiming to provide the earliest diagnosis and treatment to those the most at risk of NH while still providing adequate monitoring and follow up to those deemed less at risk. As 6 in 10 neonates <38 weeks developed NH within 24 h, preventive PT for neonates <35 weeks, and for those born 35 to 37 weeks with risk factors, was implemented using a simple checklist (S1 File). For those born 38 weeks or more the checklist aims to alert the staff to neonates with an increased risk to develop NH so they can plan for the follow-up.

The strength of the results presented relies on a large dataset of routinely collected clinical and laboratory variables and a low proportion of missing information. Notwithstanding, there are limitations: neonates with potentially high NH risk (e.g., born by caesarean section after a rupture of membranes ≥18 hours) were excluded and results likely underestimate NH incidence and proportion of severe jaundice. Exclusive breastfeeding was universal including in preterm neonates and its association with NH could not be measured. No score of acute bilirubin encephalopathy was used preventing international comparison [41]. G6PD deficiency was evaluated using a qualitative phenotypic method (FST test, [27]) and the effect of G6PD genotypes on risk of NH was not evaluated. The checklist developed requires access to simple laboratory services e.g., for blood grouping, HCT or TSB and would not be helpful to reduce the risk for severe jaundice in homeborn neonates, a major risk for exchange transfusion in rural Myanmar [42] where the government supports auxiliary midwives [43] and two in three births still take place at home.

## Conclusions

In this resource-limited setting with a dedicated special baby care unit but not intensive care nor timely access to exchange transfusion, this epidemiological study identified a high NH prevalence, 1 in 4 developing within 24 hours of life. Active screening likely lowered severe case incidence but mortality due to encephalopathy was still 1 in 10, and this is probably an underestimate. Guidelines were modified towards early NH identification and treatment and adequate follow up of those at risk of late NH. Sgaw Karen have the highest risk of NH according to ethnicity but are internationally known as Burmese refugees. The increased risk of NH in neonates identified as G6PD deficient by fluorescent spot test most likely underestimate the risk in female neonates and further research is currently evaluating this. This study demonstrates that mortality and potentially crippling morbidity from severe NH can be managed in a resource limited setting that can provide screening for TSB and offer phototherapy based on guidelines.

## Supporting information

S1 Fig Differences in total serum bilirubin median levels at 24 h, 48 h, and 72 h of life among neonates born at a gestational age ≥38 weeks who would later develop NH and those who remained NH free. Each boxplot delineates the 25^th^ and 75^th^ quartiles with the median represented by the straight bold line. The outliers are represented by the dots. The grey boxplots represent data of neonates who would remain NH free and the yellow boxplots of neonates who would later develop NH.
(TIF)

S1 Table General characteristics of 1283 neonates by time of occurrence of first NH episode within the first 7 days of life (168 hours). Data are presented as n (%).
(DOCX)

S2 Table Uni- and multivariable analysis of potential risk factors for developing NH in the first week of life (within 168 hours) using Cox proportional hazard mixed model clustering by site, n = 1283. Data are presented as n (%). ^a^Adjusted for Primigravida, Pre-eclampsia or eclampsia, Rupture of membrane ≥ 18 h, Delayed cord clamping, Gestational age <38 weeks, Birth bruising or haematoma, Sgaw Karen ethnicity, G6PD deficiency (by FST), ABO incompatibility, Severe infection at 0–24 h, Weight loss ≥7% at 24 h, and Polycythaemia at 24 h. Gender was not included because it was highly correlated with G6PD deficiency (by FST). ^**b**^ Rupture of membrane ≥18 h n = 1258, Birth bruising or haematoma n = 1282, Ethnicity (Sgaw Karen) n = 1250, Positive coombs test n = 1234.
(DOCX)

S1 File NH risk factors checklist & recommendations. (DOCX)

## References

[1] Mwaniki MK, Atieno M, Lawn JE, Newton CR. Long-term neurodevelopmental outcomes after intrauterine and neonatal insults: a systematic review. Lancet. 2012;379(9814):445–52. Epub 2012/01/17. doi: 10.1016/S0140-6736(11)61577-8 . 22244654 PMC3273721

[2] Le Pichon JB, Riordan SM, Watchko J, Shapiro SM. The Neurological Sequelae of Neonatal Hyperbilirubinemia: Definitions, Diagnosis and Treatment of the Kernicterus Spectrum Disorders (KSDs). Curr Pediatr Rev. 2017;13(3):199–209. Epub 2017/08/18. doi: 10.2174/1573396313666170815100214 . 28814249

[3] Olusanya BO, Teeple S, Kassebaum NJ. The Contribution of Neonatal Jaundice to Global Child Mortality: Findings From the GBD 2016 Study. Pediatrics. 2018;141(2). doi: 10.1542/peds.2017-1471 . 29305393

[4] Slusher TM, Vaucher YE. Management of neonatal jaundice in low- and middle-income countries. Paediatr Int Child Health. 2020;40(1):7–10. Epub 2019/12/27. doi: 10.1080/20469047.2019.1707397 . 31875773

[5] Neonatal jaundice: clinical guideline. National Collaborating Centre for Women’s and Children’s Health. Royal College of Obstetricians and Gynaecologists; London. 2010.

[6] Thielemans L, Trip-Hoving M, Landier J, Turner C, Prins TJ, Wouda EMN, et al. Indirect neonatal hyperbilirubinemia in hospitalized neonates on the Thai-Myanmar border: a review of neonatal medical records from 2009 to 2014. BMC Pediatr. 2018;18(1):190. Epub 2018/06/14. doi: 10.1186/s12887-018-1165-0 . 29895274 PMC5998587

[7] Bancone G, Chu CS, Somsakchaicharoen R, Chowwiwat N, Parker DM, Charunwatthana P, et al. Characterization of G6PD genotypes and phenotypes on the northwestern Thailand-Myanmar border. PLoS One. 2014;9(12):e116063. Epub 2014/12/24. doi: 10.1371/journal.pone.0116063 . 25536053 PMC4275285

[8] Bancone G, Gilder ME, Chowwiwat N, Gornsawun G, Win E, Cho WW, et al. Prevalences of inherited red blood cell disorders in pregnant women of different ethnicities living along the Thailand-Myanmar border. Wellcome Open Res. 2017;2:72. Epub 2017/11/29. doi: 10.12688/wellcomeopenres.12338.2 . 29181452 PMC5686509

[9] Hoogenboom G, Thwin M, Velink K, Baaijens M, Charrunwatthana P, Nosten F, et al. Quality of intrapartum care by skilled birth attendants in a refugee clinic on the Thai-Myanmar border: a survey using WHO Safe Motherhood Needs Assessment. BMC pregnancy and childbirth. 2015;15(1):17. Epub 2015/02/06. doi: 10.1186/s12884-015-0444-0 . 25652646 PMC4332741

[10] Boel M, Carrara VI, Rijken M, Proux S, Nacher M, Pimanpanarak M, et al. Complex Interactions between soil-transmitted helminths and malaria in pregnant women on the Thai-Burmese border. PLoS neglected tropical diseases. 2010;4(11):e887. doi: 10.1371/journal.pntd.0000887 . 21103367 PMC2982827

[11] Carrara VI, Hogan C, De Pree C, Nosten F, McGready R. Improved pregnancy outcome in refugees and migrants despite low literacy on the Thai-Burmese border: results of three cross-sectional surveys. BMC pregnancy and childbirth. 2011;11:45. doi: 10.1186/1471-2393-11-45 . 21679475 PMC3142536

[12] Hashmi AH, Solomon N, Lee SJ, Min AM, Gilder ME, Wiladphaingern J, et al. Nutrition in transition: historical cohort analysis summarising trends in under- and over-nutrition among pregnant women in a marginalised population along the Thailand-Myanmar border from 1986 to 2016. Br J Nutr. 2019;121(12):1413–23. Epub 2019/04/23. doi: 10.1017/S0007114519000758 . 31006391

[13] Dubowitz LM, Dubowitz V, Goldberg C. Clinical assessment of gestational age in the newborn infant. J Pediatr. 1970;77(1):1–10. Epub 1970/07/01. doi: 10.1016/s0022-3476(70)80038-5 . 5430794

[14] Rijken MJ, Lee SJ, Boel ME, Papageorghiou AT, Visser GH, Dwell SL, et al. Obstetric ultrasound scanning by local health workers in a refugee camp on the Thai-Burmese border. Ultrasound Obstet Gynecol. 2009;34(4):395–403. doi: 10.1002/uog.7350 . 19790099 PMC3438883

[15] Guideline: Delayed Umbilical Cord Clamping for Improved Maternal and Infant Health and Nutrition Outcomes. WHO Guidelines Approved by the Guidelines Review Committee. Geneva. 2014. 26269880

[16] Turner C, Carrara V, Aye Mya Thein N, Chit Mo Mo Win N, Turner P, Bancone G, et al. Neonatal intensive care in a Karen refugee camp: a 4 year descriptive study. PLoS One. 2013;8(8):e72721. doi: 10.1371/journal.pone.0072721 . 23991145 PMC3749980

[17] Thielemans L, Trip-Hoving M, Bancone G, Turner C, Simpson JA, Hanboonkunupakarn B, et al. Neonatal Hyperbilirubinemia in a Marginalized Population on the Thai-Myanmar Border: a study protocol. BMC Pediatr. 2017;17(1):32. doi: 10.1186/s12887-017-0798-8 . 28109243 PMC5251236

[18] American Academy of Pediatrics Subcommittee on H. Management of hyperbilirubinemia in the newborn infant 35 or more weeks of gestation. Pediatrics. 2004;114(1):297–316. doi: 10.1542/peds.114.1.297 . 15231951

[19] Ozdek S, Kul M, Baris Akcan A, Cekmez F, Aydemir G, Aydinoz S, et al. The effect of the pre-pregnancy weight of the mother and the gestational weight gain on the bilirubin level of term newborn. J Matern Fetal Neonatal Med. 2016;29(15):2434–7. Epub 2015/09/29. doi: 10.3109/14767058.2015.1086743 . 26413983

[20] Consultation WHOE. Appropriate body-mass index for Asian populations and its implications for policy and intervention strategies. Lancet. 2004;363(9403):157–63. Epub 2004/01/17. doi: 10.1016/S0140-6736(03)15268-3 . 14726171

[21] Janet S, Carrara VI, Simpson JA, Thin NWW, Say WW, Paw NTM, et al. Early neonatal mortality and neurological outcomes of neonatal resuscitation in a resource-limited setting on the Thailand-Myanmar border: A descriptive study. PLoS One. 2018;13(1):e0190419. doi: 10.1371/journal.pone.0190419 . 29304139 PMC5755780

[22] Turner C, Carrara V, Thien NA, Paw NM, Rijken M, McGready R, et al. Changes in the body weight of term infants, born in the tropics, during the first seven days of life. BMC Pediatr. 2013;13(1):93. doi: 10.1186/1471-2431-13-93 . 23768173 PMC3686593

[23] Villar J, Cheikh Ismail L, Victora CG, Ohuma EO, Bertino E, Altman DG, et al. International standards for newborn weight, length, and head circumference by gestational age and sex: the Newborn Cross-Sectional Study of the INTERGROWTH-21st Project. Lancet. 2014;384(9946):857–68. Epub 2014/09/12. doi: 10.1016/S0140-6736(14)60932-6 . 25209487

[24] Johnston M, Landers S, Noble L, Szucs K, Viehmann L. Breastfeeding and the use of human milk. Pediatrics. 2012;129(3):e827–41. Epub 2012/03/01. doi: 10.1542/peds.2011-3552 . 22371471

[25] Noel-Weiss J, Courant G, Woodend AK. Physiological weight loss in the breastfed neonate: a systematic review. Open Med. 2008;2(4):e99–e110. Epub 2008/01/01. . 21602959 PMC3091615

[26] Basu S, Kaur R, Kaur G. Hemolytic disease of the fetus and newborn: Current trends and perspectives. Asian J Transfus Sci. 2011;5(1):3–7. Epub 2011/05/17. doi: 10.4103/0973-6247.75963 . 21572705 PMC3082712

[27] Thielemans L, Gornsawun G, Hanboonkunupakarn B, Paw MK, Porn P, Moo PK, et al. Diagnostic performances of the fluorescent spot test for G6PD deficiency in newborns along the Thailand-Myanmar border: A cohort study. Wellcome Open Res. 2018;3:1. Epub 2018/03/20. doi: 10.12688/wellcomeopenres.13373.1 . 29552643 PMC5829521

[28] McGready R, Paw MK, Wiladphaingern J, Min AM, Carrara VI, Moore KA, et al. The overlap between miscarriage and extreme preterm birth in a limited-resource setting on the Thailand-Myanmar border: a population cohort study. Wellcome Open Res. 2016;1:32. Epub 2019/01/08. doi: 10.12688/wellcomeopenres.10352.3 . 30607368 PMC6305214

[29] Slusher TM, Zamora TG, Appiah D, Stanke JU, Strand MA, Lee BW, et al. Burden of severe neonatal jaundice: a systematic review and meta-analysis. BMJ Paediatr Open. 2017;1(1):e000105. Epub 2018/04/11. doi: 10.1136/bmjpo-2017-000105 . 29637134 PMC5862199

[30] Mason E, McDougall L, Lawn JE, Gupta A, Claeson M, Pillay Y, et al. From evidence to action to deliver a healthy start for the next generation. Lancet. 2014;384(9941):455–67. Epub 2014/05/24. doi: 10.1016/S0140-6736(14)60750-9 . 24853599

[31] Maisels MJ, Bhutani VK, Bogen D, Newman TB, Stark AR, Watchko JF. Hyperbilirubinemia in the newborn infant > or = 35 weeks’ gestation: an update with clarifications. Pediatrics. 2009;124(4):1193–8. Epub 2009/09/30. doi: 10.1542/peds.2009-0329 . 19786452

[32] Olusanya BO, Kaplan M, Hansen TWR. Neonatal hyperbilirubinaemia: a global perspective. Lancet Child Adolesc Health. 2018;2(8):610–20. doi: 10.1016/S2352-4642(18)30139-1 . 30119720

[33] Wouda EMN, Thielemans L, Darakamon MC, Nge AA, Say W, Khing S, et al. Extreme neonatal hyperbilirubinaemia in refugee and migrant populations: retrospective cohort. BMJ Paediatr Open. 2020;4(1):e000641. Epub 2020/06/17. doi: 10.1136/bmjpo-2020-000641 . 32537522 PMC7264833

[34] Bhutani VK, Wong RJ, Stevenson DK. Hyperbilirubinemia in Preterm Neonates. Clin Perinatol. 2016;43(2):215–32. Epub 2016/05/29. doi: 10.1016/j.clp.2016.01.001 . 27235203

[35] Okwundu CI, Okoromah CA, Shah PS. Cochrane Review: Prophylactic phototherapy for preventing jaundice in preterm or low birth weight infants. Evid Based Child Health. 2013;8(1):204–49. doi: 10.1002/ebch.1898 . 23878128

[36] Tyson JE, Pedroza C, Langer J, Green C, Morris B, Stevenson D, et al. Does aggressive phototherapy increase mortality while decreasing profound impairment among the smallest and sickest newborns? J Perinatol. 2012;32(9):677–84. Epub 2012/06/02. doi: 10.1038/jp.2012.64 . 22652561 PMC3558278

[37] Castillo A, Grogan TR, Wegrzyn GH, Ly KV, Walker VP, Calkins KL. Umbilical cord blood bilirubins, gestational age, and maternal race predict neonatal hyperbilirubinemia. PLoS One. 2018;13(6):e0197888. Epub 2018/06/02. doi: 10.1371/journal.pone.0197888 . 29856776 PMC5983417

[38] Boskabadi H, Rakhshanizadeh F, Zakerihamidi M. Evaluation of Maternal Risk Factors in Neonatal Hyperbilirubinemia. Arch Iran Med. 2020;23(2):128–40. Epub 2020/02/16. . 32061076

[39] Bentz MG, Carmona N, Bhagwat MM, Thimmig LM, Saleh J, Eke U, et al. Beyond "Asian": Specific East and Southeast Asian Races or Ethnicities Associated With Jaundice Readmission. Hosp Pediatr. 2018;8(5):269–73. Epub 2018/04/06. doi: 10.1542/hpeds.2017-0234 . 29618489

[40] Refugee Resettlement and Movement Management [Website]. IOM Thailand [cited 2021 March 3]. https://thailand.iom.int/refugee-resettlement-and-movement-management.

[41] Olusanya BO, Osibanjo FB, Ajiboye AA, Ayodele OE, Odunsi AA, Olaifa SM, et al. A neurologic dysfunction scoring protocol for jaundiced neonates requiring exchange transfusion. J Matern Fetal Neonatal Med. 2018;31(7):888–94. doi: 10.1080/14767058.2017.1300650 . 28320216 PMC6166872

[42] Cavallin F, Trevisanuto D, Thein A, Booth A, Arnolda G, Kumara D, et al. Birthplace is a risk factor for exchange transfusion in outborn infants admitted for jaundice in Myanmar: a case-control study. J Matern Fetal Neonatal Med. 2020;33(9):1526–31. Epub 2018/11/09. doi: 10.1080/14767058.2018.1521796 . 30407090

[43] Than KK, Morgan A, Pham MD, Beeson JG, Luchters S. Determinants of knowledge of critical danger signs, safe childbirth and immediate newborn care practices among auxiliary midwives: a cross sectional survey in Myanmar. BMJ Open. 2017;7(6):e017180. Epub 2017/07/07. doi: 10.1136/bmjopen-2017-017180 . 28679678 PMC5734551

