# Supplementary figures and images for: High levels of pathological jaundice in the first 24 hours and neonatal hyperbilirubinaemia in an epidemiological cohort study on the Thailand-Myanmar border
Source: PLoS One. 2021 Oct 7;16(10):e0258127. doi: 10.1371/journal.pone.0258127 (PMC8496801; doi:10.1371/journal.pone.0258127)

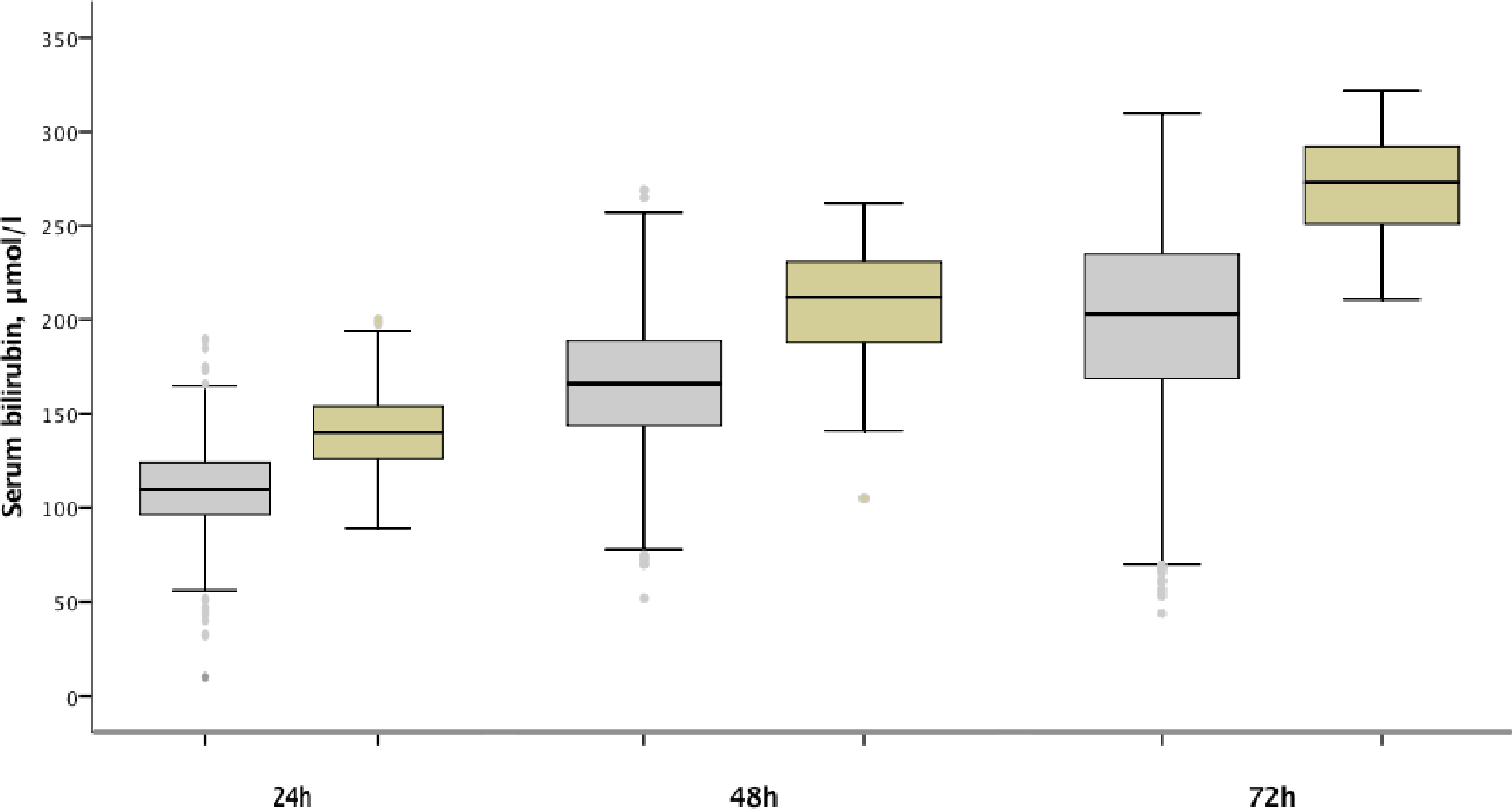

Supplement: S1 Fig — Each boxplot delineates the 25th and 75th quartiles with the median represented by the straight bold line. The outliers are represented by the dots. The grey boxplots represent data of neonates who would remain NH free and the yellow boxplots of neonates who would later develop NH. (TIF) [file pone.0258127.s001.tif]
