# Supplementary material for: High levels of pathological jaundice in the first 24 hours and neonatal hyperbilirubinaemia in an epidemiological cohort study on the Thailand-Myanmar border
Source: PLoS One. 2021 Oct 7;16(10):e0258127. doi: 10.1371/journal.pone.0258127 (PMC8496801; doi:10.1371/journal.pone.0258127)
Supplement: S1 Table — Data are presented as n (%). (DOCX) [file pone.0258127.s002.docx]

**S1 Table. General characteristics of 1283 neonates by time of occurrence of first NH episode within the first 7 days of life (168 hours)**

|  | Neonates with NH within 168 hours of life | | | Neonates without NH within 168 hours of life (n=964) |
| --- | --- | --- | --- | --- |
|  | Early NH: within 48 hours  (n=172) | Late NH:  48-168 hours  (n=147) | Overall  (n=319) |  |
| Maternal Characteristics | | | | |
| Literacy (cannot read) | 59 (34) | 45 (31) | 104 (33) | 346 (36) |
| Primigravida | 80 (47) | 61 (41) | 141 (44) | 296 (31) |
| Pre-eclampsia or eclampsia | 14 (8) | 2 (1) | 16 (5) | 15 (2) |
| Obstetric characteristics | | | | |
| Rupture of membranes ≥18h ^a^ | 18/165 (11) | 12/142 (8) | 30/307 (10) | 52/951 (5) |
| Delayed cord clamping | 136 (79) | 123 (84) | 259 (81) | 854 (89) |
| Neonatal Characteristics | | | | |
| Gestational age (<38 weeks) | 111 (65) | 19 (13) | 130 (41) | 29 (3) |
| Birth bruising or haematoma ^a^ | 11 (6) | 11 (7) | 22 (7) | 29/ 963 (3) |
| Ethnicity (Sgaw Karen) ^a^ | 80/169 (47) | 81/142 (57) | 161/311 (52) | 351/939 (37) |
| Gender (male) | 102 (59) | 85 (58) | 187 (59) | 482 (50) |
| G6PD deficiency (by FST) | 30 (17) | 19 (13) | 49 (15) | 41 (4) |
| ABO incompatibility | 37 (22) | 22 (15) | 59 (19) | 138 (14) |
| Positive Coombs test ^a^ | 10/157 (6) | 3/140 (2) | 13/297 (4) | 31/937 (3) |
| Clinical events in first 24h of life | | | | |
| Severe infection at 0-24 h of life | 14 (8) | 10 (7) | 24 (8) | 39 (4) |
| Weight loss ≥7% at 24 h [12-30 h] of life | 7 (4) | 5 (3) | 12 (4) | 23 (2) |
| Polycythaemia (HCT ≥70%) at 24 h [12-30 h] of life | 20 (12) | 13 (9) | 33 (10) | 81 (8) |

Data are presented as n (%).

^a^ Rupture of membrane ≥18 h n=1258, Birth bruising or haematoma n=1282, Ethnicity (Sgaw Karen) n=1250, Positive Coombs test n=1234.
