# Supplementary material for: High levels of pathological jaundice in the first 24 hours and neonatal hyperbilirubinaemia in an epidemiological cohort study on the Thailand-Myanmar border
Source: PLoS One. 2021 Oct 7;16(10):e0258127. doi: 10.1371/journal.pone.0258127 (PMC8496801; doi:10.1371/journal.pone.0258127)
Supplement: S2 Table — Data are presented as n (%). aAdjusted for Primigravida, Pre-eclampsia or eclampsia, Rupture of membrane ≥ 18 h, Delayed cord clamping, Gestational age <38 weeks, Birth bruising or haematoma, Sgaw Karen ethnicity, G6PD deficiency (by FST), ABO incompatibility, Severe infection at 0–24 h, Weight loss ≥7% at 24 h, and Polycythaemia at 24 h. Gender was not included because it was highly correlated with G6PD deficiency (by FST). b Rupture of membrane ≥18 h n = 1258, Birth bruising or haematoma n = 1282, Ethnicity (Sgaw Karen) n = 1250, Positive coombs test n = 1234. (DOCX) [file pone.0258127.s003.docx]

**S2 Table. Uni- and multivariable analysis of potential risk factors for developing NH in the first week of life (within 168 hours) using Cox proportional hazard mixed model clustering by site, n=1283**

| Characteristics | Neonates without NH  (n=964) | Neonates with NH  (n=319) | Univariable analysis | | Multivariable analysis ^a^ | |
| --- | --- | --- | --- | --- | --- | --- |
|  |  |  | HR [95% CI] | p-value | HR [95% CI] | p-value |
| Maternal Characteristics | | | | | | |
| Literacy (cannot read) | 346 (36) | 104 (33) | 0.87 [0.69, 1.10] | 0.234 |  |  |
| Primigravida | 296 (31) | 141 (44) | 1.77 [1.42, 2.21] | <0.001 | 1.74 [1.37, 2.20] | <0.001 |
| Pre-eclampsia or eclampsia | 15 (2) | 16 (5) | 2.86 [1.73, 4.74] | <0.001 | 1.41 [1.09, 1.82] | 0.010 |
| Obstetric characteristics | | | | | | |
| Rupture of membranes ≥18h ^b^ | 52/951 (5) | 30/307 (10) | 1.71 [1.17, 2.50] | 0.005 | 1.93 [1.32, 2.83] | 0.001 |
| Delayed cord clamping | 854 (89) | 259 (81) | 0.59 [0.45, 0.79] | <0.001 | 0.94 [0.68, 1.31] | 0.726 |
| Neonatal Characteristics | | | | | | |
| Gestational age (<38 weeks) | 29 (3) | 130 (41) | 12.0 [9.60, 15.0] | <0.001 | 13.40 [10.60, 16.90] | <0.001 |
| Birth bruising or haematoma ^b^ | 29/ 963 (3) | 22 (7) | 2.18 [1.41, 3.36] | <0.001 | 2.10 [1.31, 3.37] | 0.002 |
| Ethnicity (Sgaw Karen) ^b^ | 351/939 (37) | 161/311 (52) | 1.43 [1.10, 1.86] | 0.008 | 1.45 [1.11, 1.89] | 0.006 |
| Gender (male) | 482 (50) | 187 (59) | 1.36 [1.08, 1.70] | 0.008 |  |  |
| G6PD deficiency (by FST) | 41 (4) | 49 (15) | 3.23 [2.38, 4.39] | <0.001 | 3.89 [2.81, 5.38] | <0.001 |
| ABO incompatibility | 138 (14) | 59 (19) | 1.37 [1.03, 1.82] | 0.029 | 1.16 [0.87, 1.56] | 0.311 |
| Positive Coombs test ^b^ | 31/937 (3) | 13/297 (4) | 1.30 [0.74, 2.27] | 0.357 |  |  |
| Clinical events in first 24h of life | | | | | | |
| Severe infection at 0-24 h of life | 39 (4) | 24 (8) | 1.87 [1.23, 2.84] | 0.003 | 1.41 [0.89, 2.22] | 0.142 |
| Weight loss ≥7% at 24 h [12-30 h] of life | 23 (2) | 12 (4) | 1.53 [0.86, 2.72] | 0.152 | 1.66 [0.93, 2.97] | 0.089 |
| Polycythaemia (HCT ≥70%) at 24 h [12-30 h] of life | 81 (8) | 33 (10) | 1.31 [0.91, 1.88] | 0.148 | 1.45 [1.00, 2.12] | 0.052 |

Data are presented as n (%).

^a^ Adjusted for Primigravida, Pre-eclampsia or eclampsia, Rupture of membrane ≥ 18h, Delayed cord clamping, Gestational age <38 weeks, Birth bruising or haematoma, Sgaw Karen ethnicity, G6PD deficiency (by FST), ABO incompatibility, Severe infection at 0-24 h, Weight loss ≥7% at 24 h, and Polycythaemia at 24 h. Gender was not included because it was highly correlated with G6PD deficiency (by FST).

**^b^** Rupture of membrane ≥18 h n=1258, Birth bruising or haematoma n=1282, Ethnicity (Sgaw Karen) n=1250, Positive Coombs test n=1234.
