# Supplementary material for: High levels of pathological jaundice in the first 24 hours and neonatal hyperbilirubinaemia in an epidemiological cohort study on the Thailand-Myanmar border
Source: PLoS One. 2021 Oct 7;16(10):e0258127. doi: 10.1371/journal.pone.0258127 (PMC8496801; doi:10.1371/journal.pone.0258127)
Supplement: S1 File — (DOCX) [file pone.0258127.s004.docx]

**
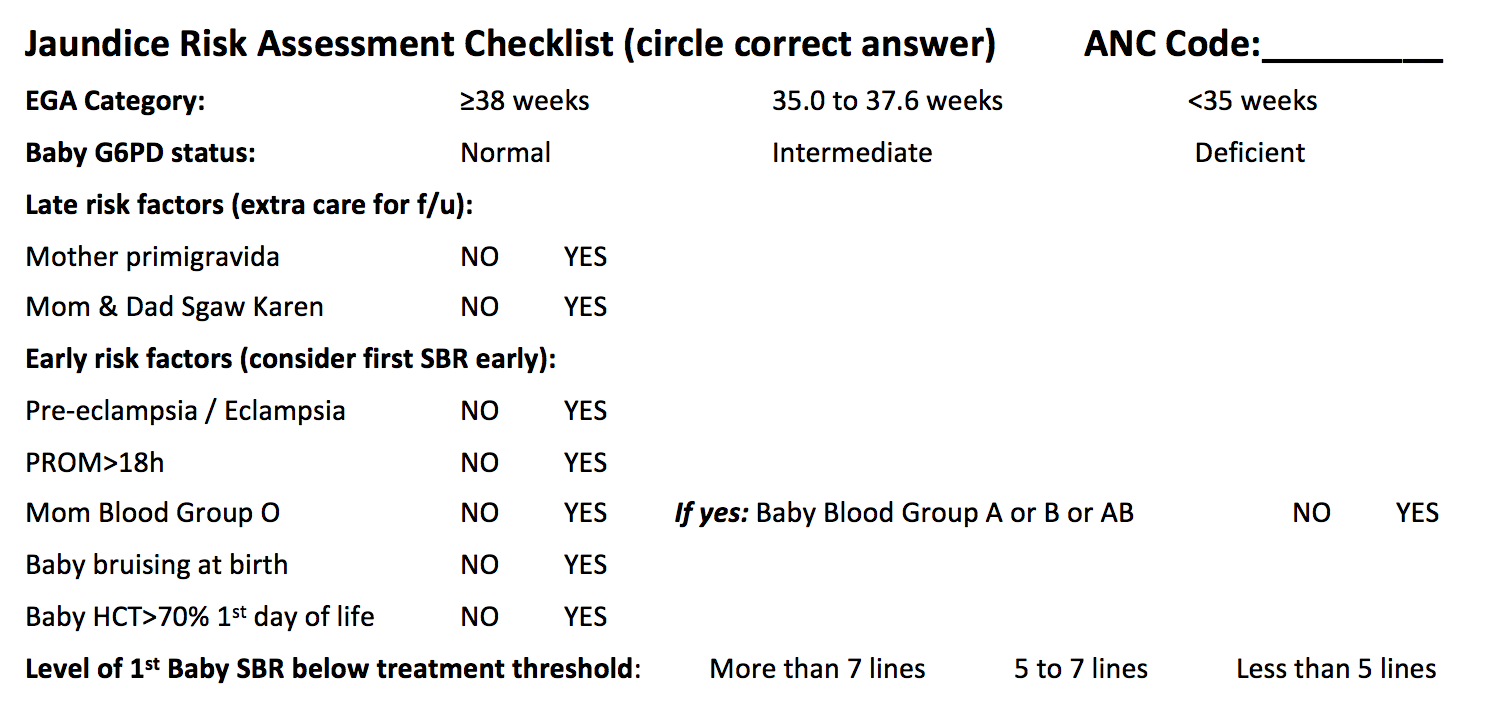
S1 File. NH risk assessment checklist and recommendations**

| **EGA<35+0 weeks** |
| --- |
| **RISK: EXTREME**  Risk is independent of G6PD status or additional risk factors  Send to SCBU once the infant is stabilized post-delivery/post first 4 hours of observation  *(with or without completed assessment risk checklist or blood taken)*  Start PT prophylaxis if SBR below treatment threshold (see protocol) or PT treatment as necessary |

| **EGA 35+0 to 37+6 weeks** | | |
| --- | --- | --- |
| **RISK: VERY HIGH TO HIGH**  Check in PNC: G6PD status AND risk factors | | |
| **G6PD DEFICIENT**  **or INTERMEDIATE** | **G6PD NORMAL**  **≥ 3 RISK FACTORS** | **G6PD NORMAL**  **0-2 RISK FACTORS**  EARLY RF PRESENT:  Keep in PNC 48h  **+** |
| Send to SCBU once the infant is stabilized post-delivery/post first 4 hours of observation  *(with or without a first SBR done)*  Start PT prophylaxis if SBR below treatment threshold (see protocol) or start PT treatment as necessary | | 1^st^ SBR: ≤12h after birth  F/u SBR: 3-12h later if SBR <5 lines below the threshold (see infant condition)  At least daily SBR until D/C, check slope  After D/C: SBR every 48h until D7 |

| **EGA ≥38+0 weeks** | | | |
| --- | --- | --- | --- |
| **RISK: MODERATE TO LOW** | | | |
| **G6PD DEFICIENT or INTERMEDIATE**  **1^st^ SBR time depends on risk factors** | | **G6PD NORMAL**  **Stay in PNC depends on risk factors** | |
| **≥ 1 EARLY RF**  Keep in PNC 48h  1^st^ SBR **≤12h** after birth  **+** | **0 RF or LATE RF only**  1^st^ SBR **≤24h** after birth  **+** | **≥ 1 EARLY RF**  Keep in PNC 48h  1^st^ SBR **≤24h** after birth  **+** | **0 RF or LATE RF only**  1^st^ SBR **before** D/C from PNC  **+** |
| F/u SBR: 3-12h later if SBR **<7 lines** below the threshold (see infant condition)  At least daily SBR until D/C, check slope  After D/C: SBR every 48h until D7 & report results in baby LEMA | | F/u SBR: 3-12h later if SBR <5 lines below the threshold (see infant condition)  After D/C: SBR and infant condition after 2-3 days, check slope & report results in baby LEMA  Counsel mother about danger signs at home!! | |
